# PReS-FINAL-2163: Disease activity in a juvenile idiopathic arthritis population after 5 years follow-up

**DOI:** 10.1186/1546-0096-11-S2-P175

**Published:** 2013-12-05

**Authors:** R Marques, F Ramos, AF Mourão, F Martins, H Canhão, JE Fonseca, JA Pereira da Silva

**Affiliations:** 1 Rheumatology Department, Lisbon Academic Medical Centre, Lisbon, Portugal; 2 Rheumatology Research Unit, Instituto de Medicina Molecular - Faculdade de Medicina da Universidade de Lisboa, Lisbon, Portugal; 3 Rheumatology, Portuguese Society of Rheumatology, Lisbon, Portugal

## Introduction

The main goal of juvenile idiopathic arthritis (JIA) treatment is to achieve a long-term remission or, at least, low levels of disease activity.

## Objectives

To evaluate disease activity, focusing on achieving inactive disease (ID) or minimal disease activity (MDA), based on Juvenile Arthritis Disease Activity Score (JADAS) score, after 5 years follow-up.

## Methods

A cross sectional study at the 5^th ^year of follow-up from a JIA patients cohort, diagnosed between 2000-2008 was carried out. Treatment strategy followed the Portuguese recommendations for the treatment of JIA. The data were collected using Reuma.pt (Portuguese Register of Rheumatic Diseases). At the 5^th ^year JADAS-27 was evaluated and used to identify patients who met the preliminary criteria for ID or MDA: score of 1 for ID and 2 and 3.8, for MDA, respectively, for patients with oligo and polyarticular involvement. For applying the JADAS score, children with rheumatoid factor (RF)-positive polyarthritis, RF-negative polyarthritis, or extended oligoarthritis were included in the polyarthritis group and the oligoarthritis group included children with persistent oligoarthritis. Children with JIA that were classified in the remaining ILAR categories were assigned to the poly or oligoarthritis group based on the number of joints affected during disease course (>4 or <4, respectively). Cutoffs for acceptable symptom state ranged from 3.2 to 5.4 for parents.

## Results

Eighty one JIA patients were identified with a follow-up of at least 5 years. Fourteen patients were excluded due to loss for follow-up and 2 developed criteria for systemic erythematous lupus. Forty four were female (68%), mean age at diagnosis 7.7 ± 4.9 years and mean follow-up 7.6 ± 2.6 years (minimum 5, maximum 13). From the 65 patients, 28(43.1%) had persistent oligoarthritis, 11(16.9%) RF-negative polyarthritis, 11(16.9%) enthesitis related arthritis, 8(12.3%) extended oligoarthritis, 3(4.6%) RF-positive polyarthritis, 2(3.1%) psoriatic arthritis and 2(3.1%) systemic JIA. Seventy three percent were on methotrexate, 11% on sulphasalazine and 23% on biologics (53%etanercept, 20%adalimumab, 20%Infliximab, 7%anakinra). At the 5^th ^year the mean JADAS was 0.78 for persistent oligoarthritis, 2.1 for extended oligoarthritis, 4.8 for RF-positive polyarthritis, 5.4 for RF-negative polyarthritis, 0.75 for psoriatic arthritis, 0.5 for enthesitis related arthritis and 0.3 for systemic JIA. In our population 68% had ID criteria at the 5th year after diagnosis and 7.7% had MDA. From those patients who had ID criteria, 50% had achieved that status after 2 years of follow-up and 81% after 3 years. From the 11 cases who had neither ID nor MDA, 7 were poly JIA (4 RF negative and 3 positive). All of them except 3 had acceptable symptoms stated by the parents.

## Conclusion

At the 5th year of follow-up, with current management strategies in daily clinical practice, around 68% of our JIA patients had achieved ID by JADAS criteria. However, for almost 17% of the patients (mostly of the polyarticular subtype), the current treatment strategies were insufficient to reach this goal, suggesting that these patients need a more aggressive treatment strategy.

## Disclosure of interest

None declared.

